# Diagnosing Temporal Lobe Epilepsy in the Emergency Department: A Case Report

**DOI:** 10.5811/cpcem.43526

**Published:** 2025-11-22

**Authors:** Ashlynn Felker, Jason Tanner

**Affiliations:** University of Utah Health, Department of Emergency Medicine, Salt Lake City, Utah

**Keywords:** seizure, temporal lobe epilepsy, electroencephalogram, déjà vu, case report

## Abstract

**Introduction:**

Temporal lobe epilepsy is a form of focal epilepsy that originates in the temporal lobes, often presenting with a variety of symptoms including altered consciousness, automatisms, and focal seizures with or without impaired awareness. Given such a diversity of manifesting symptoms, recognizing temporal lobe epilepsy in the emergency department (ED) can be challenging. Early identification is crucial for appropriate management, including timely initiation of antiepileptic therapy and differentiation from other neurological emergencies.

**Case Report:**

A 50-year-old male with no prior history of seizures or neurological conditions presented to the ED after experiencing unusual sensations that had begun three days earlier. The patient described an intermittent sensation of warmth rising from his pelvis to his head, accompanied by an experiential déjà vu-like feeling he described as “dream reenactment.” His episodes had become progressively more frequent, occurring approximately once every 90 minutes within the first 24 hours, with two instances of brief loss of consciousness. Diagnostic workup, including neurology consultation and an electroencephalogram in the ED, revealed a 19-second, non-motor focal seizure originating from the left anterior temporal region, consistent with temporal lobe epilepsy. Magnetic resonance imaging showed no acute structural abnormalities. The patient was diagnosed with temporal lobe epilepsy, started on lacosamide, and discharged from the ED.

**Conclusion:**

This case underscores the importance of recognizing temporal lobe epilepsy in the ED, particularly in patients with recurrent episodes of altered consciousness or unusual sensory experiences. Prompt diagnosis and treatment are critical to preventing further seizures and improving quality of life.

## INTRODUCTION

Temporal lobe epilepsy is a common form of focal epilepsy that originates in the temporal lobes of the brain, leading to recurrent seizures that can present in various clinical forms, ranging from focal onset seizures with or without impaired awareness to more generalized convulsions.^1^–^3^ Given that the semiology of temporal lobe epilepsy can include various symptoms such as unusual sensory experiences, altered consciousness, automatisms, and postictal confusion, recognizing this neurological disorder in the emergency department (ED) can be challenging. Prompt identification of temporal lobe epilepsy is crucial, however, for timely initiation of antiepileptic drug therapy, prevention of further seizure activity, and evaluation for possible underlying causes, such as brain lesions or structural abnormalities.^4^ Additionally, distinguishing between epilepsy-related seizures and other potential neurological emergencies, such as stroke or infection, is essential for reducing morbidity and improving patient outcomes in the ED setting.^5^ Therefore, emergency clinicians must be equipped with the knowledge and skills to accurately identify and manage temporal lobe seizures.

## CASE REPORT

A 50-year-old male with no significant past medical history presented to the ED following an episode of altered mental status. The patient had no prior medical problems and no prior history of seizures, traumatic brain injury, or neurological conditions. His symptoms had begun suddenly three days prior to ED presentation when he noticed the onset of unusual sensations. He described experiencing a rising sensation, accompanied by a conscious “dream reenactment” and a sense of warmth that started in his pelvis and radiated upward to his head. These episodes were initially brief but progressively increased in frequency and duration. The patient had previously sought care at a local urgent care and was diagnosed with vasovagal syncope, but he presented to the ED due to persistent and worsening symptoms.

The episodes were characterized by staring spells with preserved awareness. He reported mild confusion after some, but not all, episodes. The episodes typically lasted 30–45 seconds, occurring randomly throughout the first two days, and became more frequent, occurring every 90 minutes during the day leading up to presentation. He also reported two instances of loss of consciousness, both of which were brief (less than 5 minutes) and only one of which had been witnessed. During his ED visit he experienced another episode of staring with preserved awareness, which was witnessed by ED staff; it was accompanied by a brief episode of sinus bradycardia of 40–50 beats per minute, with no other vital sign changes. There were no observed motor symptoms, either focal or generalized, and no pupillary changes noted during the episode.

On examination, the patient was alert, oriented, and able to engage in conversation. Neurological findings were otherwise unremarkable, with no signs of focal deficits or significant abnormality on the initial assessment. Given his persistent and worsening symptoms, and an episode witnessed in the ED, neurology was consulted. An electroencephalogram (EEG) was performed in the ED, which showed evidence of a 19-second, non-motor focal seizure originating from the left anterior temporal region. The seizure activity was suggestive of left anterior temporal lobe epileptogenicity, consistent with temporal lobe epilepsy. Magnetic resonance imaging of the brain was also obtained while in the ED, which showed no acute structural abnormalities.

The clinical presentation, together with the EEG findings, led to a new diagnosis of temporal lobe epilepsy. The left anterior temporal lobe was identified as the likely origin of the seizure activity, and the absence of prior seizure history and structural brain abnormalities further supported this diagnosis. The patient was initiated on intravenous lacosamide with a loading dose of 200 mg and was prescribed a maintenance dose of 100 mg orally twice daily. Outpatient neurology was arranged for ongoing management and adjustment of his treatment regimen as necessary, and the patient was discharged home.

*CPC-EM Capsule*

What do we already know about this clinical entity?

*Temporal lobe epilepsy (TLE) often presents with subtle sensory or experiential symptoms that can mimic syncope, anxiety, or migraine.*

What makes this presentation of disease reportable?

*Rapidly escalating focal seizures with preserved awareness and ictal bradycardia illustrate an uncommon presentation of new-onset TLE.*

What is the major learning point?

*Recurrent déjà vu, visceral warmth, or dreamlike sensations may represent focal seizures rather than benign or psychogenic events.*

How might this improve emergency medicine practice?

*Recognizing atypical seizure presentations allows prompt neurology consultation or referral, preventing misdiagnosis and delayed care.*

## DISCUSSION

Temporal lobe epilepsy is a significant cause of focal seizures, yet it is often under-recognized, especially in its early stages when symptoms can be subtle or atypical.^1^,^2^ Although diagnosis is usually made during childhood and adolescence, temporal lobe epilepsy is the most common focal epilepsy in adults, representing an estimated 60% of all focal epilepsies and approximately 30% of all epilepsy cases overall. Given that the prevalence of epilepsy is approximately 1% in developed countries, temporal lobe epilepsy affects nearly 0.3% of the general population.^6^,^7^ The disorder is characterized by focal seizures originating from the temporal lobe, which may manifest as a variety of sensory or experiential phenomena, or auras, such as flushing, déjà vu, olfactory or gustatory hallucinations, epigastric rising sensations, intense emotions (such as fear), and auditory or visual hallucinations.^8^ These auras often precede a seizure by seconds or minutes. During the aura, a patient retains awareness and can describe motor, sensory, autonomic, or psychic symptoms. These symptoms are subjective. Because they are not commonly recognized as presenting symptoms of epilepsy in the ED, they can be mistaken for non-epileptic events such as migraine aura, transient ischemic attack, psychogenic non-epileptic seizures, psychiatric disorders or other neurological emergencies such as transient global amnesia, vestibular disorders or sleep disorders, and near-syncope or syncope.^8^,^9^

The typical frequency of temporal lobe seizures is highly variable, ranging from multiple episodes per day to only a few per year.^10^ The rapid onset and increasing frequency of seizures in this case—progressing to every 90 minutes over a span of three days—was more abrupt than typically seen in temporal lobe epilepsy, making this presentation particularly noteworthy. Symptoms like the “rising sensation,” “dream reenactment” or déjà vu sensation, and preserved awareness during episodes are less commonly recognized in the ED setting as symptoms of epilepsy. While ictal tachycardia is common in patients with temporal lobe epilepsy, ictal bradycardia, as was seen in this case, is less frequent and typically associated with focal seizures with impaired awareness, mimicking or coinciding with syncope and further complicating diagnosis.^11^,^12^ This highlights the importance for emergency physicians to be vigilant and consider temporal lobe epilepsy in their differential diagnosis, particularly in patients presenting with unusual, recurrent episodes of altered mental status, staring, or altered awareness or perception.

This case also underscores the differences in available resources across various ED settings, highlighting the diagnostic challenges that emerge in resource-limited environments. While this case occurred in a tertiary-care center with in-house neurology and use of conventional full-montage EEG in the ED, such resources are often not readily available in many settings, making conditions such as temporal lobe epilepsy challenging to recognize and manage. In such environments, point-of-care EEG devices may offer some diagnostic utility; however, to our knowledge, these tools have not been specifically studied or validated for the diagnosis of this neurological disorder, and further research is needed to evaluate their effectiveness in this context. Additionally, patient transfer may be considered if the patient exhibits persistently altered mental status, otherwise unexplained focal neurological deficits, or other concerns for status epilepticus. For stable patients without such concerns, local management may be appropriate with timely neurology referral arranged.^2^,^13^

Specialist consultation also informs treatment decisions. In this case, lacosamide was selected based on neurology input. However, levetiracetam is also a safe and effective first-line option for new-onset temporal lobe epilepsy, as it is broadly available and is generally well tolerated. Studies suggest comparable efficacy and side-effect profiles between levetiracetam and lacosamide; therefore, treatment should be individualized based on patient factors such as comorbidities and potential drug interactions.^2^,^14^

Furthermore, temporal lobe epilepsy has been associated with increased intensity and frequency of psychiatric disorders such as anxiety and depression.^15^ Early recognition in the ED can lead to timely diagnosis and treatment, potentially preventing complications such as injury, cognitive decline, and psychological distress, while also improving long-term outcomes for patients.

## CONCLUSION

This case illustrates a typical presentation with atypical frequency of newly diagnosed temporal lobe epilepsy in an adult, with characteristic symptoms and EEG findings of left anterior temporal lobe involvement. Early diagnosis and initiation of antiepileptic therapy are critical to controlling the patient’s seizures and improving quality of life. Regular follow-up with neurology and further diagnostic monitoring are essential to ensure appropriate management of this condition.
